# Elemental and Configural Associative Learning in Spatial Tasks: Could Zebrafish be Used to Advance Our Knowledge?

**DOI:** 10.3389/fnbeh.2020.570704

**Published:** 2020-12-17

**Authors:** Alexis Buatois, Robert Gerlai

**Affiliations:** ^1^Department of Psychology, University of Toronto Mississauga, Mississauga, ON, Canada; ^2^Department of Cell and Systems Biology, University of Toronto, Toronto, ON, Canada

**Keywords:** spatial learning, configural learning, elemental learning, relational learning, zebrafish, *Danio rerio*

## Abstract

Spatial learning and memory have been studied for several decades. Analyses of these processes pose fundamental scientific questions but are also relevant from a biomedical perspective. The cellular, synaptic and molecular mechanisms underlying spatial learning have been intensively investigated, yet the behavioral mechanisms/strategies in a spatial task still pose unanswered questions. Spatial learning relies upon configural information about cues in the environment. However, each of these cues can also independently form part of an elemental association with the specific spatial position, and thus spatial tasks may be solved using elemental (single CS and US association) learning. Here, we first briefly review what we know about configural learning from studies with rodents. Subsequently, we discuss the pros and cons of employing a relatively novel laboratory organism, the zebrafish in such studies, providing some examples of methods with which both elemental and configural learning may be explored with this species. Last, we speculate about future research directions focusing on how zebrafish may advance our knowledge. We argue that zebrafish strikes a reasonable compromise between system complexity and practical simplicity and that adding this species to the studies with laboratory rodents will allow us to gain a better understanding of both the evolution of and the mechanisms underlying spatial learning. We conclude that zebrafish research will enhance the translational relevance of our findings.

## Introduction

Spatial learning is a complex form of associative learning whereby the human or non-human animal learns and remembers the dynamic relationships among multiple environmental cues. It requires perceiving and attending to multiple stimuli (the conditioned stimuli or CS’s), the acquisition of relational information about these stimuli and its association with the reinforcer (the unconditioned stimulus, or US), and the consolidation, maintenance, and recall of the relational memory (the memory of dynamic relationships among the spatial cues). We emphasize that the association between these environmental cues and the reinforcer includes establishing association among the cues, i.e., configural or relational information processing. Spatial learning represents a unique subset of relational/configural learning in which the subject establishes its position relative to spatial cues, and using these cues, navigates towards its goal, a hypothesis first comprehensively discussed in the cognitive map theory by O’Keefe and Nadel (1978).

This type of learning may have high fitness value in rapidly changing, spatially complex, i.e., natural, environments. Spatial learning has been demonstrated in multiple contexts including migration (Winkler et al., 2014; Lindecke et al., 2019; Franzke et al., 2020), foraging (Croney et al., 2003; D’Adamo and Lozada, 2003; Buatois and Lihoreau, 2016), territoriality and reproductive behavior (Füller et al., 1983; Hardenberg et al., 2000). Not surprisingly, spatial learning is found in a variety of species from insects (Wystrach, 2018) to humans (Keller and Just, 2016; Piber et al., 2018). Nevertheless, the overwhelming majority of spatial learning studies has been conducted with rodents (e.g., Gerlai, 2002a,b).

In this review, however, we focus on a relatively novel laboratory organism, the zebrafish. First, we briefly discuss the frequently employed task, the Morris water maze. Subsequently, we extend the discussion to elemental vs. configural associative learning and memory and the implications of this distinction for the analysis of spatial learning and memory. Until this point, the review will cover data mostly obtained with rodents. Subsequently, we briefly summarize why the zebrafish, in general, may be useful in the analysis of learning and memory. This summary will be mainly provided in the context of biomedical research but will include considerations about evolutionary homology, and the power of the comparative approach. After the general discussion on the pros and cons of the use of zebrafish, we will review the small, but rapidly expanding, the literature on the cognitive capabilities of the zebrafish, with a focus on how analysis of this species may enhance our knowledge on spatial learning and memory. The review will end with discussions about possible future directions we regard important.

## The Morris Water Maze

No review about spatial learning is written without mentioning the Morris water-maze (Morris et al., 1982), a task that has become the gold standard in the analysis of neurobiological mechanisms underlying spatial learning and memory (Vorhees and Williams, 2006). It is an aversive conditioning task, in which spatial (and non-spatial) associative learning and memory performance of rats or mice are tested. The rodent is required to escape from an unpleasant environment, the cold water of a large circular tank. An escape platform is positioned in the maze just below the water surface onto which the rodent can climb and thus escape from the water (Vorhees and Williams, 2006). The experimental subject cannot identify the position of the platform using local landmarks adjacent to the platform, as there are none, and the platform location is also undiscoverable using olfactory cues. Escape, i.e., finding and climbing onto the safe platform, is assumed to be only possible if the animal swimming in the maze learns the dynamic relationships among the external visual cues, and remembers the platform location relative to these cues. The task has become particularly popular since it was recognized that performance in it may depend upon the hippocampus (e.g., Morris et al., 1982; Gerlai et al., 1995; Redish and Touretzky, 1998; Dong et al., 2013), a mammalian brain structure known for its role in relational learning and consolidation and recall of relational memory (Scoville and Milner, 1957; O’Keefe and Nadel, 1978; Eichenbaum, 1992; Dupret et al., 2008; Konkel et al., 2008). The Morris water maze task also has a non-spatial version in which the platform is marked by a visible cue and external spatial cues are obscured (Morris et al., 1982; Gerlai et al., 1995; Cain et al., 2006; Wagner et al., 2013), a control task that may account for performance factors unrelated to spatial learning and memory. Another reason for the popularity of the task is that it is deceptively simple (Gerlai and Clayton, 1999). Nevertheless, although it is practically simple to run, the task turned out to be rather complex both in terms of the brain functions it taps into and in terms of the interpretation of results it provides, questions we return later (Gerlai, 2001).

The study of spatial learning has been conducted on multiple fronts, only one of which has been the analysis of underlying behavioral processes. The other major line of research has concerned neurobiological mechanisms that underly learning in this task. A fundamental discovery in these analyses was the identification of “place cells” in the hippocampus, large pyramidal neurons that were found to respond to specific locations when the animal was moving around in mazes (O’Keefe, 1979). Although the original terminology “place cell” remains in use as of today, the role of these pyramidal neurons has been discovered to be not only in responding to specific spatial locations (the place field) but also to different kinds of relational information associated with space, time and cues of all modalities, including the direction of movement of the animal, proportions, olfactory cues, sequence of events, near-future decisions, as well as all possible combinations of these and other types of information as well (Eichenbaum, 2000; Save et al., 2000; for a review also see Sweatt, 2010). In fact, these cells are not about the place at all, and should be called “multimodal relational information processing cells” instead, a term that is more in-line with not only what we know about the mechanistic aspects of the hippocampus, but also what the configural association theory, first proposed by Sutherland and Rudy (1989), predicted.

By now the mechanistic understanding of spatial learning extends well beyond place cells. This advancing knowledge includes numerous synaptic mechanisms, hundreds of molecular components of neuronal plasticity and biochemical pathways in hippocampal neurons, as well as the functional roles of neuroanatomical structures other than the hippocampus (e.g., see Sweatt, 2010). Nevertheless, despite advances in our understanding of neural mechanisms underlying spatial learning, what behavioral strategies animals use in spatial or relational learning tasks are still debated. Moreover, the main hypotheses focus on spatial learning based upon a global scene and rarely on the question of how this scene is perceived, and how its elements are used in establishing spatial memory.

## Behavioral Strategies in a Spatial Learning Task

**Figure 1:**
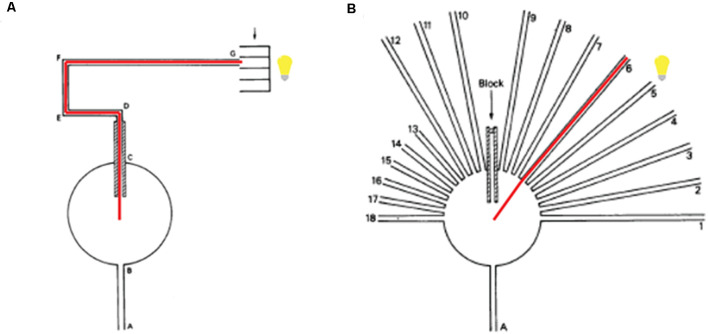
Tolman’s spatial learning setup. **(A)** During pre-training, rats entered into a circular arena passing by A and B. Once in the arena, the only possible path is to follow C, a pathway with 18 inches walls that obstructed the rat’s vision. Following the path from D to G (surrounded by transparent walls), the rat’s goal, G, was a feeding box. A light was placed in such a way that the path from D to G was illuminated. The red line indicates the only path possible for the rat. **(B)** Following training in maze **(A)**, rats were tested in the sunburst (maze **B**), the same location as during training but now equipped with 18 possible paths directly opening from the circular arena. During this test, the original training path was blocked. Out of the 18 possible arms (choices), only one, the sixth arm, pointed at the specific position of the feeding box. Out of 56 previously trained rats, 20 rats chose this shortest route, indicated by red. Figure modified from Tolman et al. (1946).

Although the neural bases underlying spatial learning has been well explored during the last decades, especially in rodents, the question of what behavioral strategies the experimental subject employs in a spatial task remains debated. The results demonstrate that experimental subjects can identify a specific position in the test arena by using different surrounding cues. However, different hypotheses have been developed as to how the animal accomplishes this. The first of these, originally described by Tolman in 1948, is the cognitive map hypothesis. According to this theory, rats develop spatial maps beyond learning the specific path they took in the past to reach their goal. In other words, they map their surroundings and can use this map dynamically. To verify his theory, Tolman (1948) used the sunburst maze (Figure 1A), in which he trained rats to walk through the maze following an experimenter controlled predetermined path from, say, point A to point G, the latter being the location of food reward. Upon completion of this training, rats were tested in the same test environment (Figure 1B), but now the test maze had 18 arms open only one of which led directly to the target. If the rat learned the specific path chosen by the experimenter during its training, associative learning theory predicts that the rat should follow this same path, even when other path options allowing the animal to reach its goal faster were also available. However, if the rat learned the spatial map of the area in which the training occurred, the cognitive map theory suggests it should be able to choose the most optimal path (the fastest route) leading to its goal, which would be different from the originally trained path. Tolman found a substantial proportion of his experimental rats to choose according to the latter strategy, and thus obtained the first piece of evidence that rats may be able to build a spatial map and may be able to dynamically use this map to optimize their navigation towards a specific spatial goal. Later, the interpretation of “dynamic spatial navigation” was questioned (Tolman et al., 1946; see O’Keefe and Nadel, 1978) as Tolman utilized light to illuminate the maze, and the rats could have used this single salient cue to guide themselves.

This question, i.e., whether the experimental subject uses a single cue vs. the dynamic relationships of multiple cues in their navigation is the cornerstone of the problems we discuss in this review. Briefly, the problem has become known as the question of elemental vs. configural learning, a vexing conundrum we return shortly. Nevertheless, since Tolman’s original publication, an overwhelming number of studies have found evidence for animals being able to learn the dynamic relationships among visuospatial cues and use them in their navigation. The evidence was obtained using different paradigms and a variety of species (e.g., O’Keefe and Nadel, 1978; Cheeseman et al., 2014; Gilroy and Pearce, 2014; Toledo et al., 2020).

Moreover, mechanistic discoveries were also in line with the cognitive map hypothesis. For example, *in vivo* multi-electrode recording from hippocampal CA1-region pyramidal neurons showed that these neurons fire action potentials only when the animal (most often rats and more recently mice too) were at a particular location within their environment or test apparatus (O’Keefe and Nadel, 1978; Moser et al., 2015). These pyramidal neurons, as mentioned above, now are known to encode all sorts of relational information about stimuli of all modalities, directional information, spatial proportions, temporal information, and so on (Eichenbaum, 1992, 2000; Ranck, 2005; Moser et al., 2008; Dudchenko et al., 2019; Shinder and Taube, 2019; Trettel et al., 2019; also see Sweatt, 2010 for a comprehensive account). Importantly, neurons serving such functions have been identified not just in the traditional laboratory rodent, but also in bats (de Cothi and Spiers, 2017; Omer et al., 2018), and perhaps more surprisingly even in fish (Canfield and Mizumori, 2004), which do not possess a brain area that would resemble the structure of the mammalian hippocampus.

Although the above findings all favor the cognitive map hypothesis, other hypotheses have also been proposed. One of them is the mental snapshot hypothesis (Collett and Cartwright, 1983; Arolfo et al., 1994; Lee and Kim, 2018). According to this hypothesis, the subject takes a snapshot of the visual cues surrounding its goal in space, then, it travels until the mental snapshot and the current view it observes match. The fundamental difference between the cognitive map theory and the snapshot hypothesis is that for the latter to work, the subject must be able to observe and remember a multitude of snapshots and compare them with multiple actual scenes as it travels across the landscape/test environment. Thus, the issue with the snapshot hypothesis is that it inherently requires huge memory capacity, yet it does not provide the subject with the dynamic flexibility afforded by the cognitive map hypothesis. Furthermore, it also does not fit what we know about the anatomy and functioning of the CNS, e.g., the role of the hippocampal formation in spatial learning we briefly mentioned above.

Another alternative hypothesis to explain how animals may navigate and find their target in space can be categorized as “non-spatial” strategies. These have been studied and discussed mostly in the context of spatial tasks developed for rodents, and do have substantial empirical support. For example, in the water-maze, thigmotactic behavior, i.e., swimming at a given small distance from the wall of the water tank has been observed and found explaining a large proportion of results with genetically manipulated mice (Lipp and Wolfer, 1998). Thigmotaxis can lead to apparently excellent learning performance, i.e., finding the target platform hidden underneath the water surface fast, if the rat or the mouse learns the appropriate distance at which the platform is positioned relative to the wall of the maze, a problem that has plagued transgenic mouse research (Lipp and Wolfer, 1998). A somewhat similar non-spatial strategy has been described in the Barnes-maze, a circular elevated platform with several escape holes at the perimeter, only one of which is safe to climb into for the rodent (e.g., Riedel et al., 2018). In this spatial learning tasks, experimenters observed that after a few trials, animals started to perform “chaining,” i.e., going from one hole to the next until they found the safe hole. The common feature of these strategies is that they bypass the need to learn complex relational information about visuospatial cues, i.e., they do not require the establishment of a cognitive map. In line with this argument is the finding that rodents with abnormal hippocampal function can use these strategies and reach their goal in these spatial tasks, despite their inability to form a cognitive map, albeit the alternative strategy usually leads to suboptimal performance compared to the spatial strategy of hippocampally unimpaired animals (e.g., Lipp and Wolfer, 1998; Riedel et al., 2018).

The last non-spatial strategy we consider here is the possibility that the subject may associate the target location (goal) with a single cue, a landmark, or a very limited number of nearby cues (Pearce, 2009; Packard and Goodman, 2013). This type of associative learning is called elemental learning, as opposed to the relationship-based configural learning required for the establishment of a cognitive map. Elemental learning was recognized as a major complication for the analysis of spatial learning in rodents (e.g., Kim and Fanselow, 1992; Phillips and LeDoux, 1992; Gerlai, 1998, 2001), a conceptually simple idea, but one which represents a difficult empirical problem.

## Elemental vs. Configural Association in Spatial Learning

The reason why elemental learning posed serious complications for scientists is that it allowed rodents even with hippocampal dysfunction to achieve relatively good performance in spatial tasks. Thus, elemental learning by the subject made it difficult for the experimenter to detect genetic or pharmacological manipulation induced defects in hippocampal function using spatial tasks. The problem was particularly recognizable in an often-employed spatial task, the context and cue-dependent fear conditioning developed for rodents (Kim and Fanselow, 1992; Phillips and LeDoux, 1992; Gerlai, 1998). Elemental learning requires acquiring and remembering the association between a maximum of two cues, the conditioned stimulus (CS), a landmark, for example, and the unconditioned stimulus (US), the reinforcer or target goal, thereby bypassing the need to learn and remember spatial relationships among multiple cues that would define the target location (George et al., 2001). Spatial learning, on the other hand, is considered a configural form of learning (Sutherland and Rudy, 1989) since it involves the integration of many different cues, i.e., learning and remembering the three-dimensional dynamic spatial relationships among these cues. Although the focus of most studies has been on visual cues, by now we know that configural learning can be multimodal, and, in addition to visual cues (Croney et al., 2003; D’Adamo and Lozada, 2003; Sturz et al., 2010), animals may employ olfactory (Lavenex and Schenk, 1995; Rossier and Schenk, 2003) or even magnetic cues (Sandberg et al., 2000; Newton and Kajiura, 2020) in their spatial or configural learning. However, even if one assumes that the animal perceives cues of only a single modality, say vision, there may be other complex aspects of the spatial learning task one has to consider.

For example, in insects, some evidence suggests that in visuospatial navigation, the animal may use a mixed strategy by initially using navigation based on path-integration (configural memory) and subsequently switching to an elemental strategy looking for prominent proximal landmarks near the target (see the review Collett and Collett, 2002). In rodents, evidence for a similar strategy has been obtained. In the context of the Morris water maze, for instance, rats can find the platform in absence of distal cues, even though the performance was better when both distal and proximal visual cues were provided (Cain et al., 1997). A mixed strategy may certainly make sense when the animal needs to traverse large distances to reach its goal. In a more restricted environment, such as the confines of the laboratory where spatial cues are not abundant, such mixed strategy may be employed in the reversed order. That is, first the animal may swim towards a clearly identifiable landmark that predicts the rough direction towards the target, and subsequently narrows its search for its target location based upon triangulating using configural information. Considering the above, it is thus likely that proximal, or local, vs. distal or global cues may be utilized in different ways during navigation. To further complicate things, perception of cues may also need to be factored into the evaluation of spatial learning performance, as proximal and distal visual cues may be perceived with different ease and precision depending upon visual acuity (Carman and Mactutus, 2002).

The complexity of the natural or the test environment, as eluded to above, may also bias the animal towards elemental vs. configural learning strategies. For example, elemental learning may be rather sub-optimal in a large and spatially complex place in which the relationships (relative configuration) among spatial cues change dynamically as the animal moves around. It would also be the wrong strategy in an environment in which there are no landmarks proximal to the target location.

Analyses of different species have demonstrated that the distinction between elemental and configural learning is not just a behavioral method related. These forms of learning are associated with distinct underlying neurobiological processes and brain structures. For example, in rodents the hippocampus is required to solve spatial learning tasks, whereas the involvement of the hippocampus is unnecessary for the elemental association as shown by countless studies using lesioning, pharmacological or genetic manipulations to damage or disrupt the functioning of the hippocampus (O’Keefe et al., 1975; Morris et al., 1982; Sutherland et al., 1982; Alvarado and Rudy, 1995; Gerlai et al., 1995, 1998; Gerlai and Roder, 1996). Interestingly, despite their small and simple brain, bees also have a structure, called “mushroom bodies,” that are considered to be functionally analogous to the mammalian hippocampus in that they are the center of sensory integration important in configural learning in insects (Mizunami et al., 1998; Liu et al., 1999; Devaud et al., 2015). Similar to the role of the mammalian hippocampus, the bee mushroom bodies are dispensable for the simple association between sugar and odor but are crucial for configural tasks such as negative patterning (Devaud et al., 2015).

Last, we want to emphasize that the experimental manipulation, e.g., induced alteration in brain function, and the test environment/procedure employed by the experimenter interacts with each other, and this interaction is what determines the result of the study. While well accepted in principle, such interaction may pose vexing problems in empirical research. An interesting experimental example of this is the already mentioned context and cue-dependent fear conditioning. This behavioral paradigm has been used to test hippocampal lesion or targeted or natural mutation-induced disfunction. The results of these studies are particularly illuminating from our perspective. They fit into the theory of configural learning and led to the concept of foreground vs. background cues, questions we examine next.

## Elemental vs. Configural Learning in Context and Cue Dependent Fear Conditioning

The first comprehensive account of configural association theory was published by Sutherland and Rudy (1989), who conceptualized two systems, a simple association system (now called the elemental association system) and the configural association system. Their theory of configural association system was very similar to the cognitive (spatial) map theory introduced by Tolman (1948) and more comprehensively proposed by O’Keefe and Nadel (1978), but differed from it as it extended the configural aspect of the association from spatial cues to any kind of relational information. Later, indeed, the configural association system started to be used synonymously to relational processing, as originally proposed by Hirsh (1974), and relational learning and memory, as studied and discussed by Eichenbaum (1992, 2000). Perhaps the biggest accomplishment of the configural association theory, in addition to properly explaining a large body of data published previously, was that it provided empirically testable predictions. For example, Sutherland and Rudy (1989) proposed a simple learning paradigm, negative patterning, *via* manipulation of configural (relational) aspect of experimenter-controlled cues, a point to which we return later. But first, let us examine the context and cue dependent fear conditioning paradigm, which, just like the water maze, became the “gold standard” of relational learning and memory analyses.

In this aversive conditioning paradigm, rodents (mice or rats) receive a mild electric shock (US) paired with a tone cue (CS) in a particular shock chamber (context 1). The paradigm has several variations, but in the most popular one, the experimental subjects are tested the following day in two different ways. In the first test, the subject is presented with the tone cue that during training predicted the delivery of the shock, but this tone cue presentation is performed in a test chamber (context 2) different from the chamber in which the training occurred the day before (context 1). The other test is performed in the original training chamber (context 1), again without the shock, but this time also without the tone cue. In both tests, the experimenter quantifies the time mice or rats freeze (stay completely immobile), which is believed to be the measure of their fear, assumed to correlate with the strength of their fear memory. The tone test serves as a test of learning and remembering the elemental association between the tone cue (CS) and the electric shock (US). Whereas the test in the training chamber (context 1) serves what became known as the context test, i.e., the test of learning and remembering the place where the shock training occurred. This latter test is assumed to tap into configural/relational memory because the training chamber itself is represented by a collection of a potentially large number of visuospatial and other (olfactory and tactile) cues. Thus, in this respect, the test is very similar to the Morris water maze spatial learning task. Indeed, just like in that task, in the context and cue-dependent fear conditioning task too, mice or rats with disrupted hippocampal function could not perform well, i.e., showed an impaired response to context 1, the training chamber, but responded well to the tone cue. In other words, just as Sutherland and Rudy (1989) predicted, hippocampal damage disrupted configural learning and memory (response to the training chamber) but not elemental learning and memory (response to the tone cue).

Except that in some studies, surprisingly, mice and rats with hippocampal damage were found to be able to respond to the training chamber with a high level of freezing. For example, Kim and Fanselow (1992) and Phillips and LeDoux (1992) showed with rats, and Gerlai (1998) showed with mice, that when rodents received the foot-shock training in the training chamber without the associative tone cue provided, they could respond to the training chamber the next day even when their hippocampus was dysfunctional. How was this possible? How could rats and mice with disrupted hippocampal function still perform well in a configural task, like the context test? This finding went against decades of consistent results the literature was showing.

The explanation for this conundrum, however, turned out to be simple. The context-dependent fear conditioning task may be solved using elemental learning strategy under certain circumstances, and thus did not fulfill the exclusive configural task solution requirement set by Sutherland and Rudy (1989). Kim and Fanselow (1992), Phillips and LeDoux (1992), and Gerlai (1998) demonstrated that the hippocampally compromised rats or mice could respond to the training chamber the next day, but only if the previous day training did not include the presentation of the tone cue. If the tone cue was paired with the shock during training, the authors of the above studies found that rodents with impaired hippocampal function could not respond to the training chamber the next day, as expected according to the configural learning or cognitive map theories. The results obtained by Kim and Fanselow (1992), Phillips and LeDoux (1992), and Gerlai (1998) suggested that in the absence of an experimenter provided salient cue, the hippocampally compromised rodents likely turned the configural task into an elemental task. In other words, the hippocampally impaired rodents likely picked out a cue from the background (the set of cues that characterized the context) and placed it into the foreground, making it a salient single cue of the training context that could be associated with the shock using simple elemental learning. And there lies an important experimental problem for most spatial or contextual learning tasks. Although the spatial learning tasks like the Morris water maze, the Barnes maze, and the context and cue-dependent fear conditioning paradigm are practically quite simple, it may be difficult to ascertain what cues, and how, animals learn in them (Gerlai, 1998, 2001).

The configural association theory, however, does offer a solution. As Sutherland and Rudy (1989) explained, an experimenter-controlled configuration of elemental cues may be employed. The example they gave for such a paradigm is the aforementioned negative patterning task, in which a compound stimulus has a specific value, i.e., it is associated with a distinct reinforcement different from what each of the individual elements of the compound alone is associated with. A variation of such a task would be, for example, as follows: animals are presented with CS1 (e.g., high pitch tone) reinforced, say, with food (US1) and CS2 (e.g., low pitch tone) reinforced with, say, a mild electric shock (US2). However, this pattern of association is only valid if CS3 (green light, for example) is present. When CS3 is absent, and CS4 (red light) is present, CS1 predicts the electric shock and CS2 the food. That is, the pairing between the conditioned stimuli and the reinforcers, the predictive values of CS1 and CS2, are reversed. This is a classical configural-relational task because the animal must learn that what CS1 or CS2 predicts is dependent upon the presence or absence of CS3 and CS4. There are numerous configural, higher-order, conditioning tasks developed for rodents which in their main principle are the same as the negative patterning task, i.e., in which the co-presentation of elemental cues, i.e., the compound of cues, conveys the relevant information. However, such tasks have only sporadically been employed in the analysis of hippocampal function/dysfunction and some of the results have been controversial.

For example, in a brain imaging study with humans, Duncan et al. (2018) found significant hippocampal activation associated with learning configural information. However, hippocampal lesions in the rat failed to influence performance in a Spatio-temporal learning task that was contingent upon the configuration between context and time of day (Dumigan et al., 2017). In fact, based upon such failures, some have even argued that dissociation of elemental vs. configural learning processes may be impossible, both at the level of behavioral analysis and the level of neurobiological mechanisms, as the configural aspect of the task may be viewed as resulting from combining two or more single elements into a compound element that then is treated by the learning system as if it was one cue (reviewed in Honey et al., 2014), a hypothesis that is, in its essence, similar to the theory of chunking (e.g., Capaldi et al., 1986). For example, in chunking, although the given species may have an upper limit of learning and remembering five to seven items at a time, combining these items into a coherent set, allows the organism to treat the set as a unit and consequently learn and remember a lot more than just this limited number of items (Miller, 1994; Johnson, 1970).

Irrespective of these complications, however, experimental control of individual cues, i.e., manipulation of the configural information, will likely allow experimenters to better characterize how the mammalian brain works, and in what aspects of configural vs. elemental learning and memory certain brain areas and neurobiological mechanisms may be involved.

Why would anyone want to switch to zebrafish in research aimed at addressing these complex questions, given that the main advances in spatial learning and memory have been accomplished with rodents?

## Why Should We Use Zebrafish in The Analysis of Learning and Memory?

Rodents provide excellent models to explore a multitude of different behaviors and their underlying mechanisms (Ellenbroek and Youn, 2016; for a comprehensive account see Gerlai, 2018). For example, the majority of studies investigating the neurobiological mechanisms of spatial learning employ the house mouse. Nevertheless, the zebrafish (*Danio rerio*) may also be appropriate for the analysis of the above questions, and, accordingly, it is becoming a popular organism in numerous fields of biology including behavioral neuroscience, psychopharmacology and ethology too (e.g., Kalueff et al., 2013, 2014). There are several reasons for this, some of them are practical, others are more theoretical, and concern the question of evolutionary homology and the power of comparative approaches. Let us first discuss the practical advantages of the zebrafish.

The zebrafish is a small vertebrate (4 cm long when fully grown) whose husbandry is simple. Zebrafish are highly prolific (one breeding pair can produce up to 200 eggs per spawning multiple times a week), and a large number of zebrafish can be housed in small space efficiently, which also makes this species highly amenable to high-throughput screening (Granato and Nüsslein-Volhard, 1996; Castranova and Wang, 2020). Comprehensive high throughput mutagenesis or drug screens, in turn, allow efficient identification of biochemical pathways and genetic factors underlying biological phenomena. The first demonstration of the utility of such high throughput approaches came from developmental biology (e.g., Granato and Nüsslein-Volhard, 1996). Such high throughput approaches have now been performed for numerous phenotypes, but not yet for the mechanistic analysis of learning and memory in zebrafish. Nevertheless, given that thousands of genes are suspected to be involved in neuroplastic processes underlying learning and memory (Gerlai, 2020b), and given that only a fraction of these has been identified (for a comprehensive account see Sweatt, 2010), comprehensive mutagenesis screens, or drug screens, which may identify genes, gene products and/or biochemical mechanisms involved, are clearly needed, and will likely be feasible with the zebrafish (Gerlai, 2010).

One of the reasons why learning and memory tests may be efficiently used with the zebrafish in general, and in high throughput screens in particular, is that, unlike rodents, the zebrafish is a diurnal species. Diurnality means that the primary modality the zebrafish uses for perceiving stimuli is visual. Visual cues are easier to control than stimuli of other modalities, an important consideration for learning studies. The on-set, off-set, and precise localization of visual stimuli are better accomplished than with olfactory or auditory cues, for example. Also notably, given that our species uses visual cues too, numerous consumer-grade equipment and electronic products have been developed, and are cheaply and easily accessible, for the experimental scientist. Cameras, computer monitors, and software applications are all available, some of which are specifically developed for the zebrafish, with which visual cues may be presented in a controlled and precise manner (Chouinard-Thuly et al., 2017; Gerlai, 2017b).

In addition to methodological and technical aspects of zebrafish research, we emphasize the second point, we mentioned above, which should persuade scientists to use this species. Teleosts (bony fishes) possess simple brains in which numerous features (e.g., neurotransmitter systems, synaptic processes, molecular mechanisms) are evolutionarily conserved (Metscher and Ahlberg, 1999). Even at the gross anatomy level, numerous zebrafish brain structures have been identified as homologous to certain mammalian brain areas. The simplicity of this brain coupled with its evolutionary conservation, thus, allows a reductionist and, at the same time, translationally relevant approach (Gerlai, 2020a). For example, as mentioned above, certain structures in the zebrafish brain have been found homologous to mammalian counterparts. From the perspective of spatial learning, one of the most important of these is the lateral pallium, which has been argued to be the zebrafish homolog of the mammalian hippocampus (Butler, 2000; López et al., 2000b; Mueller and Wullimann, 2009; Vargas et al., 2009; also see review by Gerlai, 2017a). Functionally, this structure has also been found similar between fish and mammals, as its lesioning, for example, leads to spatial learning deficits in both these taxa (reviewed by Rodríguez et al., 2002). Nevertheless, contrary to the mammalian hippocampus, which has a complex structure and connectome (e.g., a tri-synaptic circuit), the lateral pallium of zebrafish is simple, allowing a potentially easier mechanistic understanding of spatial learning and memory at the synaptic and molecular levels of analysis (Gerlai, 2020b). We also emphasize that the simplicity of this species also means that its features are evolutionarily old, which may allow one to identify core mechanisms that are common across most vertebrate taxa (Gerlai, 2020a). Thus, comparing species like rodents and zebrafish may provide us with discoveries that translate better to humans than if we were to study one or the other laboratory species alone, a topic that has been discussed in more detail elsewhere (Gerlai, 2020a).

## Associative Learning Paradigms Using Zebrafish

The current downside of zebrafish in the analysis of learning and memory is the relative novelty of this species, i.e., the somewhat limited number of behavioral paradigms that could be used as screening or testing tools as well as the rudimentary understanding of the cognitive and mnemonic characteristics of this species (Gerlai, 2017a). For example, a simple literature search with the Web of Science “all databases” search engine using the terms “zebrafish” AND “learning” returns 1,182 entries as of the writing of this manuscript. However, an identical search but with the term “mouse” replacing “zebrafish” returns 50,442 entries. The same search but with “rat” results in 83,898. Clearly, compared to the zebrafish, rodents dominate this field. Nevertheless, by now a substantial amount of evidence has demonstrated the utility of the zebrafish in the analysis of learning and memory. Here, we provide only a succinct review of associative learning paradigms developed for the zebrafish, sampling the literature for specific representative examples without trying to be comprehensive.

Although methodologically varied, most associative learning tasks employed successfully with zebrafish fall within the category of classical conditioning tapping into elemental learning. These tasks may be subdivided in terms of the modality of the CS and, perhaps more importantly, according to the US, i.e., the reinforcer employed. Some of the first such learning tasks developed for the zebrafish used fear conditioning (Hall and Suboski, 1995a,b) in which the aversive (fear-inducing) alarm substance (chemical cue) served as the US, and visual as well as olfactory cues were employed as the CS. However, the paradigm did not gain a strong foothold, as the alarm substance employed was a complex mixture of natural substances whose dose, exact compound identity as well as on-set and offset could not be precisely controlled, and its effects thus were found variable (reviewed in Kenney, 2020; also see Speedie and Gerlai, 2008; Parra et al., 2009). Appetitive conditioning has also been employed with zebrafish. In these learning paradigms, the reinforcer is a reward, most often food (e.g., Colwill et al., 2005; Manabe et al., 2013). However, food has been found somewhat challenging in learning paradigms with the zebrafish, as these poikilothermic animals can quickly satiate on food, and thus the motivational value of the food reward can rapidly decrease as training progresses. Nevertheless, good learning performance in food rewarded classical conditioning with olfactory (Braubach et al., 2009), auditory as well as visual cues (Doyle et al., 2017) as the CS has been demonstrated with zebrafish. We also note that the latter authors designed their learning paradigm for the home tank, thereby minimizing experimenter interference, an important topic we briefly return later. An alternative reward that turned out to be quite effective and non-satiating in learning tasks has been the sight of conspecifics (Al-Imari and Gerlai, 2008; Pather and Gerlai, 2009; Qin et al., 2014). However, perhaps even more interesting from our perspective are the results that imply the possibility of configural learning in zebrafish.

## Do We Have Evidence for Configural Learning in Zebrafish?

Conditioned Place Preference (CPP) is a frequently employed paradigm in rodents. CPP is most often used to test the rewarding/reinforcing properties of drugs of abuse or other substances. However, the paradigm achieves this not by directly comparing choice or preference for the drug vs. placebo/control, but *via* a memory task. The rodent has to remember the compartment in which the US (the drug in this case) was presented previously. The spatial aspect of the task thus implies that it may be solved using configural learning. Nevertheless, CPP is not specifically designed to dissociate elemental from configural learning, and indeed often the place of drug delivery (the side, or the compartment, of the test apparatus) is marked by a salient visual cue. Irrespectively, successful CPP has been demonstrated by several studies with zebrafish, for example by Lau et al. (2006) and by Collier and Echevarria (2013). Furthermore, in a recent study, Yashina et al. (2019) concluded that zebrafish can form spatial memory based on visuospatial and geometric cues. Similarly, evidence implying, but not proving, the ability of zebrafish to employ configural/relational learning comes from a study by Kenney et al. (2017) who adopted part of the context and cue-dependent fear conditioning task originally developed for rodents. These authors found that memory of the electric shock (i.e., the fear response) was specific to the tank in which the shock training occurred and that this memory could be blocked by the NMDA-receptor antagonist, MK-801. NMDA-receptor is known to be an important molecular component of learning and memory as it acts as a coincidence detector and is crucial for synaptic plasticity, including long-term potentiation (LTP) and long-term-depression (LTD) in the hippocampus of mammals (for a comprehensive account see Sweatt, 2010). We note, however, that although suggestive for configural learning, Kenney et al. (2017) did not employ a cued task, and thus neither could dissociate context and cue-dependent memory nor could ascertain the potentially configural learning/memory dependent effect of MK801 in their zebrafish study. Thus, just like in most rodent studies, in the Kenney et al. (2017) study too, the response of zebrafish to the shock tank, the training chamber, could have been based upon elemental learning.

**Figure 2:**
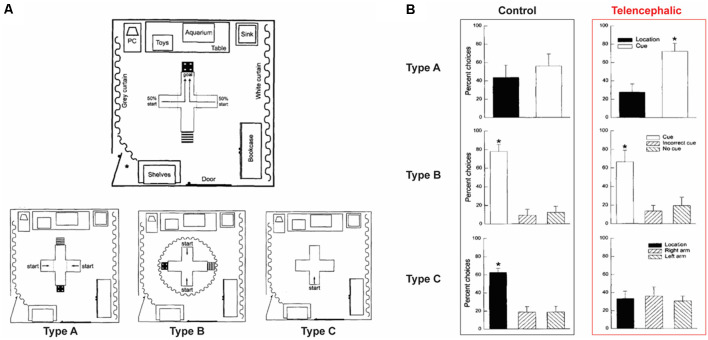
Spatial learning in goldfish. **(A)** The experimental setup was a cross-shaped tank. Fish were started either on the right or left arm (50/50). A cue card was associated with the rewarded arm during the entire training. Three types of probe tests were performed on a different group of fish that experienced the same training. *Type A*: Cue card, as well as room information, was available. *Type B*: Only the cue card was visible, room information was hidden thanks to a curtain. *Type C*: Cue card was removed, fish could only access the room information. **(B)** The bars present the percentage of choice for the location or the cue card during the probe tests. In this experiment, intact fish were used (control), as well as fish for which the telencephalon was ablated. In the probe test, similarly to zebrafish, control goldfish were able to use either the cue card or the location information. However, the telencephalon ablated fish were only able to use the cue card information. These results suggest a role of the telencephalon in configural memory retention, but not in elemental memory retention. The figure is adapted from figures in López et al. (2000a). Asterisks indicate significant (*p* ≤ 0.05) differences in the percentage of choices of the various arms.

Perhaps the strongest evidence for the ability of fish being able to acquire and remember configural information comes from studies conducted by a Brazilian group that employed spatial learning tasks analogous to the Morris water maze. Rodríguez et al. (2002) review these studies and emphasize that using lesioning of appropriate pallial areas of the goldfish brain, the results converge on one conclusion: when this brain region, which is thought to be homologous to the mammalian hippocampal formation, is lesioned, the goldfish is not able to perform well in spatial learning tasks but its performance is unimpaired in non-spatial (elemental) associative learning tasks. Similar findings were obtained by Portavella and Vargas (2005) who studied spatial learning in goldfish and analyzed the role of different telencephalic pallial systems. They showed that the medial and lateral regions of the telencephalic pallia are functionally homologous to the mammalian amygdala and hippocampus, respectively. These results are also exemplified by the studies of Broglio et al. (2010) and López et al. (2000a,b) illustrated in Figures 2A,B. Although ablation is a highly invasive procedure that may have broad confounding effects unrelated to the specific function of the ablated structure, finding specificity of the lesion effect in the spatial learning task in goldfish is highly instructive. These results with goldfish, thus, are comparable to the large body of literature that shows lesioning, or pharmacological and genetic manipulations induced dysfunction of the hippocampus of rats and mice to result in deficits in performance in the spatial but not in the non-spatial version of the Morris water-maze (O’Keefe et al., 1975; Morris et al., 1982; Sutherland et al., 1982; Alvarado and Rudy, 1995; Gerlai et al., 1995; Gerlai and Roder, 1996; Gerlai, 1998). The issue with such lesioning studies (both for rodents and fish), however, is that the apparent spatial-learning selectivity may be explained by the relative complexity of the task: a sick fish may still be able to learn in a simple elemental task but may perform badly in the complex configural task, not because of the elemental vs. configural nature of these tasks, but because of their level of difficulty.

Although the goldfish is closely related to the zebrafish (both are cyprinids), and thus a similar performance in zebrafish may be assumed, spatial learning specific performance disruption found with goldfish has not been demonstrated in zebrafish, as such lesion studies have not been conducted with the latter species. Nevertheless, the zebrafish is a good candidate for such studies, as it offers a more sophisticated manipulation than the crude lesioning methods employed in the past. Modern genome editing tools already developed for the zebrafish now allow one to specifically target regions, circuits, and even specific individual neurons in the fish brain and disrupt and/or temporally control the activity of these targeted brain areas in a non-invasive manner more specifically than lesioning could (e.g., De Santis et al., 2020; Lee et al., 2020; Tsuda, 2020), a topic we return to in the last section on future research directions.

Furthermore, although the above instructive spatial learning-specific brain lesion effects were found with goldfish, by now we do have some evidence that zebrafish too can perform well in spatial tasks. Specifically, these results demonstrated that zebrafish can perform in a spatial task just like rodents with intact hippocampal formation would (Karnik and Gerlai, 2012).

## Simultaneous Elemental and Configural Learning in A Spatial Task in Zebrafish, A Performance Resembling That of Rodents

Recall our previous discussion on the context and cue-dependent fear conditioning. One notable aspect of the findings was that in this paradigm rats (Kim and Fanselow, 1992; Phillips and LeDoux, 1992) and mice (Gerlai, 1998) could learn two distinct pieces of information simultaneously: one, the elemental association between the tone cue and the shock, and two, the association between configural cues of the training chamber and the shock, but only if the hippocampus of the rodent was intact. If the rats or mice suffered from a lesioned hippocampus, or impairment of the functioning of this structure due to mutations, they could only learn the elemental association, but not both the elemental and the configural associations.

**Figure 3:**
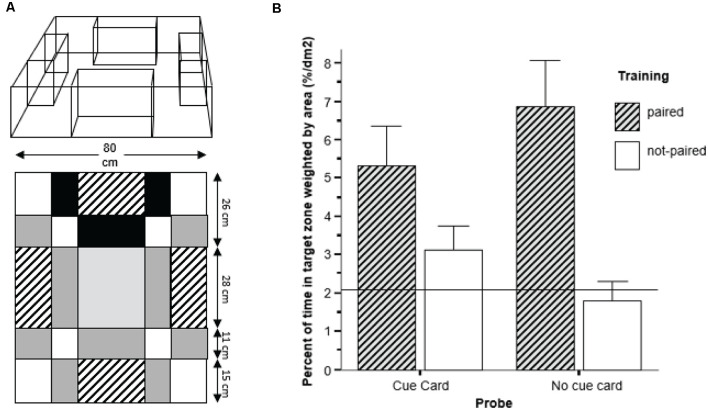
Spatial learning in zebrafish. **(A)** The experimental tank was composed of a large square tank with four stimulus tanks placed in it adjacent to each sidewall. Only one of the stimulus tanks contained five conspecifics during training and was highlighted with a red plastic cue card and always keep at the same location. The other stimulus tanks were empty and did not have a red cue card behind them. The areas with different shading served as a template for quantification of the location of the fish during training and probe test. **(B)** During the probe test, in absence of conspecifics as a reward, fish from the paired group showed a significant preference for the area marked with the red card when the cue card was presented. However, in absence of a cue card, fish showed a preference for the original location of the conspecific tank during training. These results demonstrate that zebrafish were able to use both elemental as well as configural learning strategies to simultaneously learn both the association between CS-US and location-US. Figure modified from Karnik and Gerlai (2012).

Karnik and Gerlai (2012) used a spatial learning task utilizing some of the conceptual aspects of the rodent context and cue-dependent fear conditioning task (Gerlai, 1998), but with appetitive conditioning and with zebrafish. The reward they employed was the sight of conspecifics, which previously is an efficient reinforcer in learning tasks for zebrafish (Al-Imari and Gerlai, 2008; Sison and Gerlai, 2010; Fernandes et al., 2016), perhaps because the zebrafish is a highly social, shoaling, species (Wright and Krause, 2006; Miller and Gerlai, 2007). Thus, experimental zebrafish tested alone in a learning task is expected to be motivated to seek out and try to join a shoal. Briefly, in this task, the zebrafish were required to associate a reward (presence of conspecifics, the stimulus fish) with a particular location AND a salient visual cue (Figure 3A). The results demonstrated that the experimental zebrafish could learn both the elemental association between the salient visual cue (a red cue card) and a configural association, the specific spatial position of the conspecific stimulus tank (Figure 3B), a performance that was demonstrated in rodents only if their hippocampus was fully functional (Gerlai, 1998 and references therein).

The experimental procedure and methods were relatively simple. In a large open tank, a single experimental zebrafish was allowed to swim freely. Inside the large open tank, there were four small stimulus tanks, each positioned flush to one of the four sidewalls of the tank. Three stimulus tanks were empty, and one contained live stimulus fish. The one with the stimulus fish remained in the same position and was always marked by a red cue card throughout training, i.e., throughout the twenty trials delivered over a week-long period. A day after the conclusion of this training, half of the zebrafish were tested in probe trial 1 and the other half in probe trial 2 (this ascertained avoidance of interference between the two probes). In probe trial 1, the experimental zebrafish saw no stimulus fish in any of the stimulus tanks but could see the red cue card. The position of the red cue card varied from one experimental fish to the next, i.e., it was moved randomly. This probe is equivalent to the tone cue test of the context and cue dependent fear conditioning task, and tests for the acquisition of memory of the association between the red cue card (CS) and the stimulus fish (US), i.e., elemental learning and memory. Zebrafish showed excellent performance, i.e., stayed close to the red cue card, significantly closer than to other locations. Furthermore, these trained fish also stayed significantly closer to the CS than control fish did that received training during which the red cue card and the location of the stimulus fish were randomized, the unpaired group. What is more interesting, however, is what happened during probe trial 2. In this trial, the experimental subject was released into the large open tank singly, just like in probe 1, but now both the stimulus fish and the red cue card were absent. This probe trial tested for location memory, i.e., asked whether the experimental fish would stay close to the original fixed past location of the stimulus fish, despite that they now were not there. The answer to this question was a resounding yes. The test fish spent significantly more time near this location than anywhere else, and also importantly, significantly more time than fish of the unpaired group. In other words, they remembered the spatial location, i.e., acquired configural memory.

Of course, one can still argue that these results may not fully prove the acquisition of configural memory. After all, the zebrafish could have picked out a spatial cue from the background and could have turned the configural spatial task into an elemental learning problem, similarly to what the hippocampally impaired rats (Kim and Fanselow, 1992; Phillips and LeDoux, 1992) and mice (Gerlai, 1998) did in the context and cue dependent fear conditioning. This argument is valid, but recall that when the hippocampally impaired rats or mice were presented with a salient single cue, they could not respond to the training chamber, i.e., they were unable to pick out another cue from the background. Here, zebrafish were presented with a single salient cue, the red cue card, and they did learn the association between this cue and the reward, yet they were still able to find the past location of the reward during the probe trial even though this cue was absent. That is, they performed just like rodents with intact hippocampal function. There are two possible explanations for this result. One, zebrafish, unlike rodents, can pick out a background cue from the context despite being experimentally provided with a salient foreground cue, or two, zebrafish, just like rodents, can learn elemental and configural associations simultaneously. The literature on what we know about cue salience, attention, overshadowing and associative learning suggests that the former is unlikely, and the latter, i.e., simultaneous elemental and configural learning, is the proper interpretation (e.g., Mackintosh, 1976; Esber and Haselgrove, 2011; Todd and Manaligod, 2018).

However likely is the hypothesis that zebrafish can learn elemental and configural information simultaneously, the above results still only represent indirect evidence. What would bring direct evidence is if we could specifically and systematically manipulate the stimuli that make up the configural information. There are two fundamentally distinct ways one could accomplish this. One, could control every possible visuospatial cue in a spatial task, rotate, conflict, disassemble, and slowly and systematically remove and modify such spatial cues. This would be a daunting task given that we do not exactly know what visuospatial cues zebrafish attend to and what determines the salience/importance of each of these cues in spatial learning and navigation. The second possible way would be the type of conditioning Sutherland and Rudy (1989) proposed under the term “negative patterning” and for which we gave an example at the beginning of this review. Unfortunately, negative patterning-based learning tasks have not been developed for zebrafish, and thus we do not yet have the tool to address this question. However, other paradigms one could easily adapt from mammalian studies to explore configural learning in fish may also be appropriate for the zebrafish. These include the “Delayed Matching To Sample” task (Giurfa et al., 2001; Lee et al., 2018) or the “Delayed non-Matching To Sample” task (Bruce et al., 2018; Cole et al., 2020), which may allow studying whether the subject can utilize the concept of “Sameness” or “Difference,” or at least whether it can cope with a temporal gap known to invoke the function of the hippocampus in mammals. In fact, the first example of successful demonstration of working memory in zebrafish solving a delayed matching to sample task has recently been published (Bloch et al., 2019). In rodents, the hippocampus is recruited to solve such tasks (Jagielo et al., 1990; Hampson et al., 1993). We note that in these paradigms the experimenter has control over the cues employed, and thus their configuration, i.e., the relationships among them. Furthermore, testing elemental cue discrimination is possible using the same stimuli as in the configural context, which thus represents an internal control, and would enable one to explore distinct mechanisms underlying elemental vs. configural learning.

Another way to distinguish and better understand elemental vs. configural learning is to investigate the neurobiological mechanisms that underlie these processes, the topic we examine next.

## Data on Mechanisms Underlying Elemental and Configural Learning and Memory in Zebrafish Have Started to Accumulate

As mentioned before, we do have evidence that the lateral pallium is particularly important in configural (spatial) learning, but not in elemental learning in a fish species, goldfish (Rodríguez et al., 2002), closely related to the zebrafish. We also know that a key molecular player in neuronal plasticity and hippocampal learning and memory in mammals, the NMDA-receptor, is highly conserved in zebrafish, and functions in a similar manner in zebrafish and mammals (Sison and Gerlai, 2011a,b; Kenney et al., 2017). Additional mechanistic details are also accumulating. For example, we now know that several molecular components involved in neuronal plasticity in mammals, including for example Brain-Derived Neurotrophic Factor (BDNF), Neuronal Cell Adhesion Molecule (NCAM), and synaptophysin (a synaptic protein) are all conserved and expressed in the zebrafish brain (Mahabir et al., 2018). The expression level of these proteins was found to be altered by alcohol, a substance that is known to engage molecular mechanisms underlying neuronal plasticity (Mahabir et al., 2018). Furthermore, expression levels of mRNA encoding NCAM and another NCAM, L1.1, were found to be elevated in zebrafish trained in an active avoidance task, and L1.1 was found to be involved in memory consolidation in this task in zebrafish (Pradel et al., 2000). Psychopharmacological studies have demonstrated the role of neuronal nicotinic acetylcholine receptors (nAChR’s) in spatial learning in zebrafish, similarly to their involvement in the same function in mammals (Braida et al., 2014). At the synapse level, evidence has already been obtained that zebrafish neuronal plasticity functions similarly to what we know about mammalian hippocampal pyramidal neurons, the well-known place cells. For example, high-frequency electric stimulation of the dorsal telencephalon of the zebrafish induces LTP, whereas low-frequency stimulation induces long-term depression (LTD; Wu et al., 2017), synaptic strengthening and weakening processes thought to underly the establishment of medium and long term spatial (relational/configural) memory in mammals (for comprehensive account see Sweatt, 2010). Furthermore, just like in mammals, long-term associative memory was found to be dependent upon protein synthesis (Hinz et al., 2013).

Despite these important discoveries, however, our mechanistic understanding of elemental and configural learning in fish has a lot of gaps and needs comprehensive and systematic analyses, a topic that belongs to the last question of this review: future directions of research.

## Numerous Holes in Our Current Knowledge, but A Large Number of Possibilities for The Future

There are numerous interesting possible future research lines on mechanistic as well as behavioral analysis of elemental and configural learning we foresee for zebrafish. We briefly summarize these below, starting with the behavioral side of the work, admitting that what we list here represents our personal biases.

The hypothesis that zebrafish can perform both elemental as well as configural association simultaneously during a spatial task is fascinating, and the study by Karnik and Gerlai (2012) as well as the results reviewed by Rodríguez et al. (2002) all demonstrate potentially sophisticated cognitive abilities of fish in general and perhaps of zebrafish in particular. However, as mentioned above, we would need definite proof, either by manipulation of the function of certain brain areas of zebrafish similarly to what López et al. (2000a,b) did with goldfish or by developing and using a negative patterning-like configural learning paradigm in zebrafish. Furthermore, we also know very little about the behavioral limits of learning and memory performance of the zebrafish. For example, we have no information on how long zebrafish could remember the learned information, how easy it is to extinguish or to reverse the memory, and most importantly whether memory span, extinction, and a reversal is/are distinctly different for elemental vs. configural memory.

Also, as mentioned before, likely, novel behavioral paradigms that allow the precise control of the relationships among individual stimuli, i.e., the configural aspect of the task, will need to be developed. We mentioned the negative patterning task, which utilizes controlling compound cues, and contrasted this task with the uncontrolled spatial arrangements of cues animals are required to learn in classical spatial tasks. We see these problems with past and possible future behavioral methods as arising fundamentally from two different reasons: one, practical, and two, principal, i.e., biological system function related. The practical we already mentioned: it may be difficult to precisely control, contrast, and manipulate visuospatial cues in a regular spatial task. The principle, fundamental question, however, we have not discussed. It concerns whether all pieces of relational information are created equal. For example, we do not clearly know whether temporal order of cues, spatial arrangements of cues fitting into the visual field of the animal, or spatial arrangements of cues gathered as the animal navigates through space are acquired, consolidated, and recalled in the same manner. For example, although in mammals temporally controlled presentation of elemental cues forming sequential relationships has been performed in higher-order associative conditioning successfully (e.g., De Houwer et al., 2016), such tasks have not been attempted with zebrafish. Thus, we do not understand whether these different relational/configural tasks require the same behavioral processes and have identical or overlapping underlying neurobiological mechanisms, or not. Nevertheless, we already know that visual stimulus configurations that are present in the visual field of the animal can be easily distinguished and remembered as patterns even by lower-order vertebrates such as fish (Gómez-Laplaza and Gerlai, 2020). The discrimination ability of these patterns is thought to be, at least partially, dependent upon the visual system with the analysis of the pattern/configuration starting outside of brain areas that are considered part of the configural memory processing system. This type of visual configural memory, thus, is likely distinct in terms of behavioral and mechanistic aspects from a cognitive map or classical configural memory as defined by Sutherland and Rudy (1989). The latter is established as the animal moves across the landscape and thus has to use its short term (working) and perhaps also long-term (reference) memory, unlike when the animal looks at a configuration of cues that fit into its visual field at the same time. However, systematic comparisons to prove the distinction between the processes underlying learning and remembering visual stimulus patterns and more abstract or true spatial configural patterns have not been performed. The temporal order of elemental cues forming configural information vs. the spatial arrangements of elemental cues forming configural memory also has not been systematically compared in terms of cognitive or underlying biological mechanisms. Last, multi-strategy theories, i.e., the idea of the animal using one strategy under certain circumstances and another under other circumstances, or using one strategy first and subsequently another, also need to be systematically analyzed in the future.

Another unresolved issue with zebrafish cognition research is seemingly simple but is rather important. It is a practical, methodological problem: human handling. Human handling induced stress and fear is a particularly vexing problem for zebrafish learning and memory paradigms, as zebrafish are rather sensitive to being netted out from their home tank and being placed in the novel test tank. To solve this issue, Doyle et al. (2017) have already designed a home tank-based learning paradigm, an idea not unlike the one proposed for rodent research (de Visser et al., 2006), but one which likely will make the analysis of the fine-tuned learning responses of zebrafish much easier.

The next question we consider is the mechanistic analysis of elemental and configural processes. Our understanding of biological mechanisms underlying elemental and configural association learning processes in the zebrafish has started to improve, but as our summary above showed, such studies are rather sporadic. Briefly, mechanistic studies are sorely needed at multiple levels of analysis. Once proper behavioral testing tools, i.e., high throughput phenotypical screening methods, have been developed, systematic characterization of genes, gene products, and biochemical mechanisms underlying elemental and configural learning may proceed with high speed using zebrafish (Gerlai, 2010). The mechanistic analysis will likely be aided by several modern methods already developed for the zebrafish. For example, systematic analyses of brain structures involved could be performed with detailed gene expression profiling using RT-qPCR, modern deep-sequencing methods, or single-cell PCR (Gorissen et al., 2015; Peixoto et al., 2017). Mapping c-fos staining-based neuronal activation in the brain of zebrafish is already a well-developed simple and feasible method (Chatterjee et al., 2015; Bhattarai et al., 2016). Even manipulation of specific circuits, and thus fine structural-functional mapping of relevant brain areas, is possible using optogenetic methods with the zebrafish (Tsuda, 2020). Shortly, likely, combining *in vivo* electrophysiology recording with behavior quantification may also become possible for the small zebrafish, as it has been successfully accomplished in goldfish (Canfield and Mizumori, 2004). Imaging calcium currents in live zebrafish is already a reality (Kettunen, 2020), and immobilized adult zebrafish placed in a virtual environment (Huang et al., 2020) may enable one to conduct sophisticated learning studies while recording and correlating neuronal activity using such calcium imaging methods. All these methods could be employed at different stages of a configural vs. elemental learning task (acquisition, consolidation, retention and recall) to identify dynamic functional changes in the brain specific to these cognitive processes.

The last point we discuss is whether analysis of spatial learning with fish may be useful for our understanding of the evolutionary origin of complex cognition in higher-order vertebrates like mammals. As shown by empirical studies, in spatially rich environments, non-spatial strategies lead to suboptimal performance, longer paths, and longer times to reach the goal (e.g., Wolfer and Lipp, 2004). In nature, such non-spatial strategies may be particularly maladaptive given the complexity of the abiotic and biotic natural environment (Vyssotski et al., 2002). For these reasons, it is reasonable to suppose that inability to perform a spatial strategy represents a significant adaptive disadvantage and must have been selected against during the evolutionary past of most species. In accordance with this, as we briefly reviewed, a wide range of species from primitive to complex organisms exhibit spatial learning abilities. Spatial learning, as discussed, requires acquiring, consolidating, and recalling configural information. However, this ability, once acquired through evolution, should transfer to non-spatial tasks where relational information processing is required. We mentioned abstract and temporal configuration as examples of such higher cognitive tasks. One thus may speculate that perhaps a possible evolutionary origin of complex cognition is in spatial learning, a question that is poorly understood (Morand-Ferron et al., 2016), but one which certainly merits attention in the future, and one which may be efficiently studied using such simple vertebrate organisms as the zebrafish.

## Conclusion

Spatial learning is an evolutionarily adaptive and likely ancient form of learning found across the animal kingdom. Its relational/configural information processing demands may have given rise to high cognitive functions typical of mammals, but its fundamental features and underlying core mechanisms may have their roots in lower-order organisms, such as fish. The zebrafish is an emerging new and simple laboratory organism with which the evolutionarily conserved, fundamental mechanisms of configural and elemental learning may be dissociated and better understood. Although a novice in this field, evidence already exists for the complex cognitive abilities of this little fish. With the currently existing spatial paradigms and novel configural learning tasks that may be developed in the future, and with the sophisticated neurobiological and molecular methods already available for the zebrafish, likely this species will significantly advance our knowledge of how the vertebrate brain learns and remembers relational information.

## Author Contributions

AB and RG conceptualized and wrote the review. All authors contributed to the article and approved the submitted version.

## Conflict of Interest

The authors declare that the research was conducted in the absence of any commercial or financial relationships that could be construed as a potential conflict of interest.
